# *In silico* gepotidacin target mining among 33 213 global *Neisseria gonorrhoeae* genomes from 1928 to 2023 combined with gepotidacin MIC testing of 22 gonococcal isolates with different GyrA and ParC substitutions

**DOI:** 10.1093/jac/dkae217

**Published:** 2024-07-08

**Authors:** Alexandra David, Daniel Golparian, Susanne Jacobsson, Caleb Stratton, Pham Thi Lan, Ken Shimuta, Pam Sonnenberg, Nigel Field, Makoto Ohnishi, Christopher Davies, Magnus Unemo

**Affiliations:** Institute for Global Health, Faculty of Population Health, University College London, London, UK; WHO Collaborating Centre for Gonorrhoea and Other Sexually Transmitted Infections, Department of Laboratory Medicine, Microbiology, Faculty of Medicine and Health, Örebro University, Örebro, Sweden; WHO Collaborating Centre for Gonorrhoea and Other Sexually Transmitted Infections, Department of Laboratory Medicine, Microbiology, Faculty of Medicine and Health, Örebro University, Örebro, Sweden; Department of Biochemistry and Molecular Biology, University of South Alabama, AL, USA; Hanoi Medical University, National Hospital of Dermatology and Venereology, Hanoi, Vietnam; Department of Bacteriology I, National Institute of Infectious Diseases, Tokyo, Japan; Institute for Global Health, Faculty of Population Health, University College London, London, UK; Institute for Global Health, Faculty of Population Health, University College London, London, UK; Department of Bacteriology I, National Institute of Infectious Diseases, Tokyo, Japan; Department of Biochemistry and Molecular Biology, University of South Alabama, AL, USA; Institute for Global Health, Faculty of Population Health, University College London, London, UK; WHO Collaborating Centre for Gonorrhoea and Other Sexually Transmitted Infections, Department of Laboratory Medicine, Microbiology, Faculty of Medicine and Health, Örebro University, Örebro, Sweden

## Abstract

**Objectives:**

The novel dual-target triazaacenaphthylene, gepotidacin, recently showed promising results in its Phase III randomized controlled trial for the treatment of gonorrhoea. We investigated alterations in the gepotidacin GyrA and ParC targets in gonococci by *in silico* mining of publicly available global genomes (*n* = 33 213) and determined gepotidacin MICs in isolates with GyrA A92 alterations combined with other GyrA and/or ParC alterations.

**Methods:**

We examined gonococcal *gyrA* and *parC* alleles available at the European Nucleotide Archive. MICs were determined using the agar dilution method (gepotidacin) or Etest (four antimicrobials). Models of DNA gyrase and topoisomerase IV were obtained from AlphaFold and used to model gepotidacin in the binding site.

**Results:**

GyrA A92 alterations were identified in 0.24% of genomes: GyrA A92P/S/V + S91F + D95Y/A/N (0.208%), A92P + S91F (0.024%) and A92P (0.003%), but no A92T (previously associated with gepotidacin resistance) was found. ParC D86 alterations were found in 10.6% of genomes: ParC D86N/G (10.5%), D86N + S87I (0.051%), D86N + S88P (0.012%) and D86G + E91G (0.003%). One isolate had GyrA A92P + ParC D86N alterations, but remained susceptible to gepotidacin (MIC = 0.125 mg/L). No GyrA plus ParC alterations resulted in a gepotidacin MIC > 4 mg/L. Modelling of gepotidacin binding to GyrA A92/A92T/A92P suggested that gepotidacin resistance due to GyrA A92T might be linked to the formation of a new polar contact with DNA.

**Conclusions:**

*In silico* mining of 33 213 global gonococcal genomes (isolates from 1928 to 2023) showed that A92 is highly conserved in GyrA, while alterations in D86 of ParC are common. No GyrA plus ParC alterations caused gepotidacin resistance. MIC determination and genomic surveillance of potential antimicrobial resistance determinants are imperative.

## Introduction

*Neisseria gonorrhoeae* has developed or acquired resistance to all antimicrobials used for the treatment of gonorrhoea. The last remaining options for first-line empiric treatment (ceftriaxone 0.25–1 g plus azithromycin 1–2 g, or ceftriaxone monotherapy 0.5–1 g) are threatened, with reports of ceftriaxone and azithromycin resistance increasing internationally.^1–13^ Sporadic gonococcal isolates with resistance to ceftriaxone combined with high-level resistance to azithromycin have also been identified in several countries.^13–18^ Consequently, novel antimicrobials for the future treatment of gonorrhoea are urgently needed.

The new antimicrobial gepotidacin,^19–21^ in oral dosing of 3 g plus 3 g 10–12 h later, recently showed non-inferiority compared with internationally recommended treatment (ceftriaxone 500 mg single intramuscular dose plus azithromycin 1 g single oral dose) in its Phase III randomized controlled clinical trial (RCT; ClinicalTrials.gov identifier: NCT04010539, https://clinicaltrials.gov/study/NCT04010539) for the treatment of uncomplicated urogenital gonorrhoea (https://www.gsk.com/en-gb/media/press-releases/eagle-1-phase-iii-data-show-potential-for-gepotidacin-as-a-new-oral-treatment-option-for-uncomplicated-gc/). Appropriate data regarding treatment efficacy for also extra-genital gonococcal infections, especially in the oropharynx, are imperative. Gepotidacin is a new triazaacenaphthylene bacterial topoisomerase type IIA inhibitor that inhibits DNA replication through a novel mechanism of action targeting the GyrA subunit of DNA gyrase and the ParC subunit of topoisomerase IV.^19,20^ The binding site of gepotidacin is close to, but differs slightly from that of quinolones,^22^ and *in vitro* studies have shown that *N. gonorrhoeae* has a high susceptibility to gepotidacin, with MICs ranging from 0.032 to 4 mg/L (MIC_90 _= 1 mg/L).^21,23^ Moreover, a single-step resistance selection study failed to recover gepotidacin-resistant mutants, indicating a low rate of spontaneous single-step resistance mutations.^24^ In a Phase II RCT, single gepotidacin oral doses of 1.5 and 3 g cured 97% (29/30) and 95% (37/39) of urogenital gonococcal infections, respectively.^20,21^ All the pre-treatment gonococcal isolates from the microbiological treatment failures (*n* = 3) showed a gepotidacin MIC of 1 mg/L and were fluoroquinolone-resistant with a pre-existing ParC D86N substitution (selected by the excessive use of especially ciprofloxacin in the gonorrhoea treatment in previous decades), i.e. mutation in the gepotidacin-binding site of one of the two gepotidacin targets that has been suggested to predispose for development of gepotidacin resistance.^21,23^ At test-of-cure, an A92T substitution in the other gepotidacin target (GyrA) had been selected in two of the three isolates, resulting in a high gepotidacin MIC (≥32 mg/L).^21^ Accordingly, only the ParC D86N substitution, which has been selected by fluoroquinolone use, does not significantly increase the MIC of gepotidacin, but this mutation is a prevalent stepping-stone mutation for high resistance to gepotidacin that requires also a specific mutation in the second gepotidacin target GyrA. Similar development of high-level gepotidacin resistance has been described in *Klebsiella pneumoniae*.^25^ The global prevalence of *N. gonorrhoeae* isolates with substitutions of A92 in GyrA, D86 in ParC and other potentially relevant GyrA and ParC amino acid positions has not been known.

In the present study, we investigated the global susceptibility to the novel dual-target (GyrA and ParC) antimicrobial gepotidacin through *in silico* screening of mutations in the *gyrA* and *parC* genes in 33 213 publicly available *N. gonorrhoeae* genomes and examined the gepotidacin MIC in 22 isolates with GyrA A92 alterations combined with other GyrA and/or ParC alterations.

## Materials and methods

### Gonococcal genomes

The European Nucleotide Archive (ENA) was queried (2023-02-08) for *N. gonorrhoeae* (search term Taxon: 485). Bioprojects with <10 gonococcal genomes and/or including multiple species were excluded. Gonococcal genomic sequences from 33 213 global isolates from 1928 to 2023 were included. All data were quality-controlled, and non-gonococcal sequences identified using Kraken (v1.1.1) were excluded. Pathogenwatch (https://pathogen.watch/)^26^ was used for additional quality controls and species confirmation. Isolates were *de novo* assembled using SPAdes (v3.14.1) on a CLC Genomics Workbench (v22.0.3).^27^ In total, 2062 gene alleles (1700 core and 362 accessory) were characterized in Ridom Seqsphere+ (v3.4.0) as part of a previously defined cgMLST scheme.^28^ Multiple sequence alignments of *gyrA* and *parC* alleles were extracted, and variants were called using SNP-sites (v2.5.1),^29^ and amino acid alterations subsequently characterized in the quinolone resistance-determining regions (QRDR) of each target (residues 79–101 in GyrA and 78–131 in ParC).

### Antimicrobial susceptibility testing

MICs (mg/L) of gepotidacin using the agar dilution method and ceftriaxone, cefixime, azithromycin and ciprofloxacin by the Etest (bioMérieux, Marcy-Étoile, France) were determined on GCVIT agar plates [GC agar base (GC Medium Base agar; BD Diagnostics, Sparks, MD, USA) supplemented with 1% IsoVitalex (BD Diagnostics)], as earlier described.^7,23^ The 2016 WHO *N. gonorrhoeae* reference strains were used for quality control.^30^ Only whole MIC doubling dilutions are reported.

### Molecular modelling of *N. gonorrhoeae* DNA gyrase and topoisomerase IV structures

DNA gyrase and topoisomerase IV are homologous proteins that each exist as heterotetramers. DNA gyrase is comprised of two GyrA and two GyrB subunits, while topoisomerase IV is comprised of two ParC and two ParE subunits.^31,32^ Models for *N. gonorrhoeae* GyrA, GyrB, ParC and ParE were obtained from the AlphaFold Protein Structure Database (https://alphafold.com) and the heterotetramers constructed by superimposing each subunit onto its respective subunit in the crystal structure of *Staphylococcus aureus* DNA gyrase in complex with gepotidacin (PDB: 6QTK)^33^ using Superpose in the CCP4 suite^34,35^ (Figure S1, available as Supplementary data at *JAC* Online). Coordinates corresponding to gepotidacin in the *S. aureus* structure were then docked into each model.

### Spot transformation^36^ of QRDR of *gyrA*

A segment of the *gyrA* gene was amplified using PCR as previously described.^37^ Five to ten colonies of the recipient strain (WHO F, K, L and M) were suspended in GC broth. Two spots (20 µL each) of the bacterial suspension were placed on GCVIT agar plates. The amplified product of *gyrA* (0.1 µg) suspended in Tris buffer was added to one spot, while Tris buffer without the amplified product was added to the second spot as a negative control. The plates were incubated at 35°C in a humidified 5% CO_2_-enriched atmosphere for 18–22 h. The spots were then harvested, resuspended in GC broth and subjected to 10-fold serial dilutions up to 10^6^. Selective GCVIT agar plates containing 2-fold gepotidacin MIC of recipient strain were inoculated with 100 µL from each dilution, and non-selective GCVIT agar plates without antibiotics were inoculated with 10^5^ and 10^6^ dilutions as controls. All plates were incubated at 35°C in a humidified 5% CO_2_-enriched atmosphere for 18–22 h.

## Results and discussion

The primary determinants of quinolone resistance in DNA gyrase and topoisomerase IV are mutations in the QRDRs^38,39^ of GyrA and ParC. Within the QRDR of GyrA, amino acid alterations were observed in 48.5% of the 33 213 genomes (Table S1, available as Supplementary data at *JAC* Online). The most prevalent GyrA alteration was in the main fluoroquinolone target S91, i.e. S91F/T/Y either alone (0.43%), or more commonly in combination with D95A (24.7%), D95G (20.5%), D95N (2.3%) or D95Y (0.009%). Alterations in GyrA A92, involved in gepotidacin resistance,^21,23^ were rare (0.24%): the most common being A92P + S91F + D95Y (0.17%), followed by A92P + S91F + D95A (0.03%), A92P + S91F (0.024%), A92S + S91F + D95N (0.009%), A92V + S91F + D95A (0.006%) and A92P alone (0.003%). No GyrA A92T alteration, which has previously been associated with gepotidacin resistance,^21,23^ was found (Table S1).

Within the QRDR of ParC, amino acid alterations were found in 44.4% of the genomes (Table S1). The most common ParC substitutions were S87R/N/I/C/Y alone (23.2%), but these were also observed together with E91K/Q/G/A (2.4%), S88P (1.4%) and/or G120E/R (0.3%). Alterations in ParC D86, a residue in the gepotidacin-binding site,^21,23^ were found in 10.6% of genomes. ParC D86N alone was the most prevalent (10.5%), but this mutation was also observed in combination with S87I (0.051%) and S88P (0.012%). ParC D86G alone was also found (0.006%) and combined with E91G in one isolate (0.003%) (Table S1).

We subsequently investigated the potential effects of alterations in GyrA A92 combined with other GyrA and/or ParC alterations found among the 33 213 gonococcal genomes on the MICs of gepotidacin. In the collection of gonococcal isolates at the WHO Collaborating Centre for Gonorrhoea and Other Sexually Transmitted Infections, which includes many tens of thousands of gonococcal strains, 22 clinical gonococcal isolates with known substitutions in GyrA A92 were available. In total, these 22 isolates represented 10 unique combinations of alterations in GyrA A92 and other GyrA and/or ParC alterations. Despite alterations in GyrA A92 together with other GyrA and/or ParC alterations, gepotidacin demonstrated a high *in vitro* activity against these isolates (Table 1).

**Table 1.** MIC of gepotidacin and four additional antimicrobials in 22 clinical *N. gonorrhoeae* isolates,^a^ obtained through routine diagnostics or surveillance, with GyrA A92 alterations combined with other GyrA and/or ParC substitutions

Amino acid alterations			MIC range (mg/L)					Molecular STs
GyrA	ParC	No. of isolates	Gepotidacin	Ciprofloxacin	Ceftriaxone	Cefixime	Azithromycin	MLST, NG-STAR
S91F, A92P, D95Y	S87N	6	0.25–4	4->32	0.016–0.064	0.016–0.032	0.5–1	ST8123, ST2473 (*n* = 5), ST8123, ST4592 (*n* = 1)
A92S, D95N	E91G	3	0.125–0.25	1–8	0.002–0.004	≤0.016	0.125–0.25	ST7365, ST3340 (*n* = 1), ST7822, ST3626 (*n* = 1), ST7365, ST1899 (*n* = 1)
S91F, A92P	S87R, S88P	3	0.25	4	0.064–0.125	0.25	0.5	ST7363, ST4474
A92P, D95Y	S87N	2	2	>32	0.016–0.032	0.064	0.5–1	ST8123, ST3716
S91F, A92P, D95Y	G85D, S87R	2	0.125	>32	0.032	0.032	0.5	ST10241, ST3283
S91F, A92P, D95A	S87N, E91Q	2	0.125	32	0.008	<0.016	0.064	ST1588, ST620
S91F, A92P	D86N	1	0.125	1	0.016	<0.016	0.125	ST7366, ST6039
A92P, D95A	S87N, E91Q	1	0.125	16	0.002	<0.016	0.125	ST1588, ST3675
S91F, A92P, D95A	E91G	1	0.064	8	<0.016	<0.016	0.125	ST14104, ST6040
S91F, A92P	Wild type	1	0.064	0.25	0.012	0.064	0.064	ST7363, ST4469

MLST, multi-locus sequence typing; NG-STAR, *N. gonorrhoeae* sequence typing for antimicrobial resistance.

^a^From Vietnam (*n* = 9; isolated in 2016), Japan [*n* = 4; 2009 (*n* = 1), 2014 (*n* = 2), 2016 (*n* = 1)], Italy (*n* = 3; 2019), Sweden [*n* = 2; 2018 (*n* = 1), 2022 (*n* = 1)], Austria [*n* = 2; 2016 (*n* = 1), 2021 (*n* = 1)], Norway (*n* = 1; 2017) and Pakistan (*n* = 1, 2010).

Accordingly, all isolates had a gepotidacin MIC of ≤4 mg/L (MIC range: 0.125–4 mg/L), which is mainly within the previously described gonococcal wild-type MIC distribution.^21,23^ Surprisingly, one isolate with GyrA A92P plus ParC D86N alterations, i.e. alterations in both targets for gepotidacin,^21,23^ remained susceptible to gepotidacin (MIC = 0.125 mg/L). This was further supported by the failure to increase the gepotidacin MICs in the WHO F, K, L and M gonococcal reference strains by spot transformation^36^ with GyrA P92 in repeated experiments, i.e. no mutants with increased gepotidacin MIC grew on the gepotidacin-containing selective GCVIT agar plates after repeated spot transformation.^36^

This study demonstrates that, among 33 213 publicly available global gonococcal genomes from 1928 to 2023, A92 in GyrA is highly conserved, whereas alterations of D86 in ParC are common. Accordingly, alterations in GyrA A92 were very rare (0.2%), while alterations in ParC D86 were found in 10.6% of all genomes. No GyrA A92 alterations in combination with other GyrA and/or ParC alterations resulted in phenotypic resistance to gepotidacin. Interestingly, in the previous Phase II RCT, two post-treatment gonococcal isolates with GyrA A92T plus ParC D86N alterations displayed high-level gepotidacin resistance (MIC ≥ 32 mg/L).^20,21^ However, in our study, we show that gonococcal isolates with some GyrA substitutions at A92 (A92P and A92S), even in combination with ParC D86N, remain susceptible to gepotidacin. This suggests that the GyrA A92T mutation impacts the gepotidacin interaction significantly more than other mutations at this position. Accordingly, different amino acid alterations at GyrA position A92 can result in dramatically different results on the MIC of gepotidacin. However, it should also be noted that some unknown antimicrobial resistance determinants and/or compensatory mutations in the strain with GyrA A92T plus ParC D86N alterations could be involved.

Molecular modelling of the GyrA and ParC structures provides an indication of how mutations at position 92 of GyrA and 86 of ParC might contribute to gepotidacin resistance. As shown in our model of ParC, D86 is located at the N-terminal end of an alpha helix that is sandwiched between the gepotidacin-binding and DNA-binding sites (Figure S2). Its side chain is within 4Å of the pyranopyrimidine ring of gepotidacin. Mutation of the ParC D86 residue to Asn (D86N) may lower affinity for gepotidacin due to a change in electrostatics that replaces a negative charge with a neutral one. Position 92 in GyrA is on the equivalent helix as ParC and similarly located at its N-terminal end, but this residue faces the DNA-binding site rather than the gepotidacin-binding site (Figure S3). It is on the opposite side of the helix to GyrA D90, which is equivalent to D86 in ParC. Modelling suggests that introduction of a threonine at position 92 (A92T) in GyrA (Figure S3B) could generate a new polar contact between the helix and the phosphodiester backbone of bound DNA. Potentially, this could shift the helix away from the gepotidacin-binding site and therefore weaken the contact between GyrA D90 and gepotidacin. This would not happen with the GyrA A92P mutation because this side chain is hydrophobic and would not contact the phosphodiester backbone. Molecular dynamics simulations indicate that proline does not disrupt the helical structure (data not shown).

Our work demonstrates the necessity of performing gepotidacin MIC testing to verify the effects of any GyrA or ParC mutations on the gepotidacin MICs, i.e. in addition to studies examining genomes *in silico*. This is important for novel antimicrobials such as gepotidacin and zoliflodacin, but also when investigating currently used antimicrobials. Notably, none of the 33 213 publicly available gonococcal genomes contain the GyrA A92T alteration, which, together with ParC D86N, was previously confirmed to result in a gepotidacin MIC of ≥32 mg/L.^21^ However, the ParC D86N substitution was prevalent and this alteration has been shown to predispose for development of gepotidacin resistance, i.e. to increase the resistance mutation selection in the other gepotidacin target GyrA.^21,23^ Also in a recent large European gonococcal WGS study including 1932 isolates, no GyrA A92T substitution was found, but the ParC D86N substitution was detected in 11.2% of isolates.^40^ Introduction of gepotidacin into clinical use might benefit from a rapid diagnostic point-of-care test simultaneously detecting *N. gonorrhoeae* and relevant *parC* mutations (such as ParC D86N) and *gyrA* mutations (such as GyrA A92T), to reduce gepotidacin treatment of gonococcal-negative patients or gonococcal strains that are predisposed to develop resistance to gepotidacin.

No obvious cross-resistance with the four other tested antimicrobials, including the topoisomerase II inhibitor ciprofloxacin, was identified (Table 1), which is in accordance with a previous study.^23^ However, it was surprising to recognize that six isolates lacking the GyrA S91F alteration displayed high-level resistance to ciprofloxacin (MIC = 1->32 mg/L). These isolates only contained GyrA A92P/S combined with GyrA D95A/N/Y and ParC S87N and/or E91G/Q alterations (Table 1). This raises concerns for ciprofloxacin-resistant gonococcal strains that escape detection using the ResistancePlus GC assay (SpeeDx Pty Ltd, Sydney, Australia)^41^ or similar nucleic acid amplification tests that predict resistance or susceptibility to ciprofloxacin in *N. gonorrhoeae*.^42^

### Conclusions

In conclusion, mining 33 213 publicly available global gonococcal genomes and performing gepotidacin MIC testing of 22 isolates with GyrA A92 amino acid alterations combined with other GyrA and/or ParC alterations suspected to cause resistance to gepotidacin did not identify any obvious gepotidacin-resistant gonococcal isolates and the susceptibility to gepotidacin among global gonococcal isolates is high, i.e. based on our genomic mining and previous MIC-based studies.^21,23,24^ Nevertheless, if gepotidacin is approved for the treatment of uncomplicated gonorrhoea, surveillance of susceptibility to gepotidacin (both phenotypically and genomically, especially of GyrA A92 and ParC D86) will become imperative. Moreover, it is also essential to ascribe new *gyrA* and *parC* mutations as gepotidacin-resistant mutations after adequate confirmation of their effect on the MIC of gepotidacin.

## Supplementary Material

dkae217_Supplementary_Data

## Supplementary data

Table S1 and Figures S1–S3 are available as Supplementary data at *JAC* Online.
