# Supplementary material for: In silico gepotidacin target mining among 33 213 global Neisseria gonorrhoeae genomes from 1928 to 2023 combined with gepotidacin MIC testing of 22 gonococcal isolates with different GyrA and ParC substitutions
Source: J Antimicrob Chemother. 2024 Jul 8;79(9):2221–6. doi: 10.1093/jac/dkae217 (PMC11368423; doi:10.1093/jac/dkae217)
Supplement: dkae217_Supplementary_Data [file dkae217_supplementary_data.zip › JAC-2024-0359_Revised_Supplementary figures S1-S3.docx]

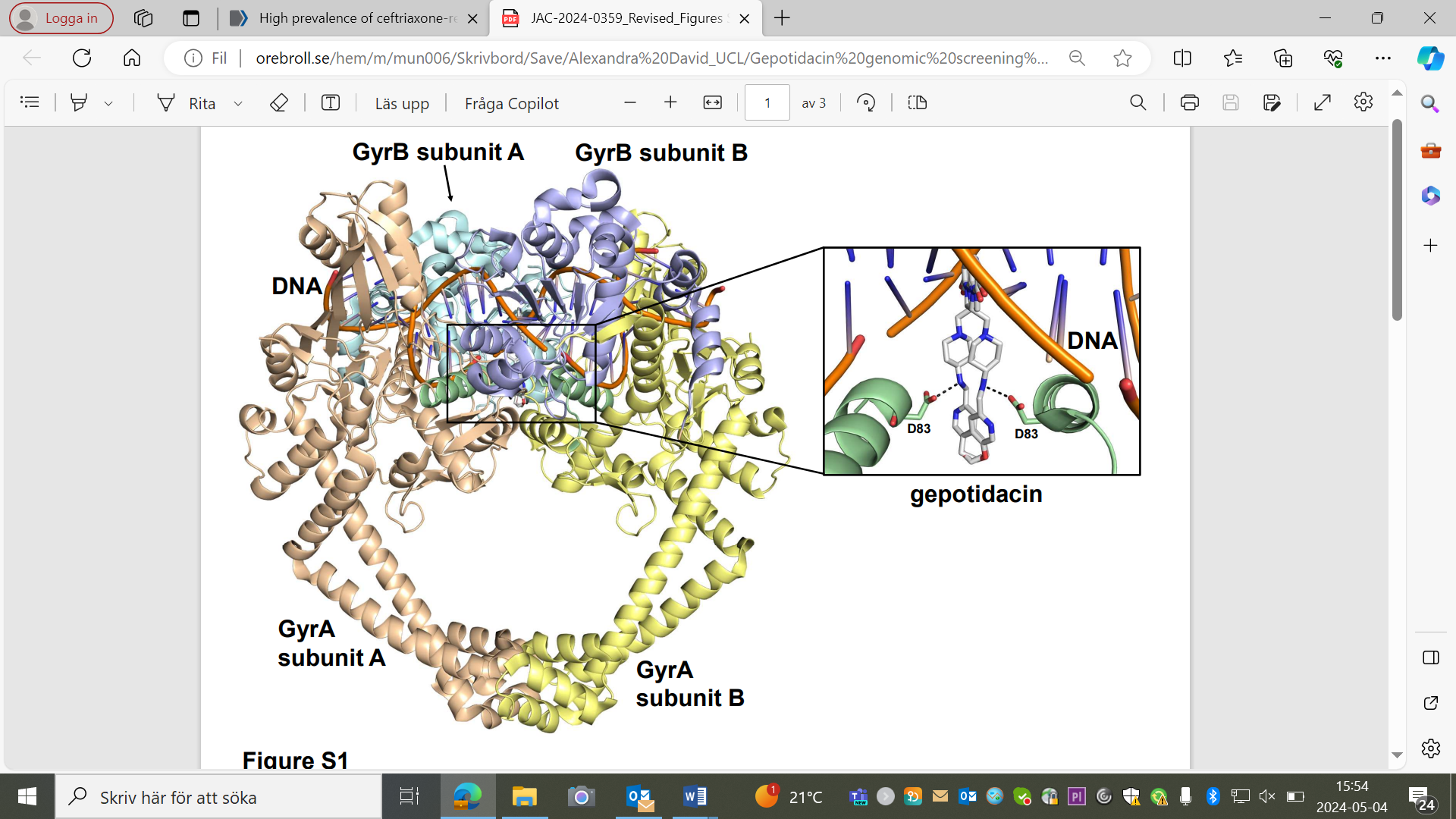


**Figure S1.** Structure of *Staphylococcus aureus* DNA gyrase in complex with gepotidacin.^33^ GyrA subunits A and B are coloured in tan and yellow, respectively, and GyrB subunits A and B in cyan and blue, respectively. The helical quinolone resistance-determining region of GyrA is shown in green. The phosphodiester backbone of DNA is orange and the bases are purple. Inset: Detail of the GyrA gepotidacin-binding site. Note two alternative conformations of gepotidacin are present in the structure.


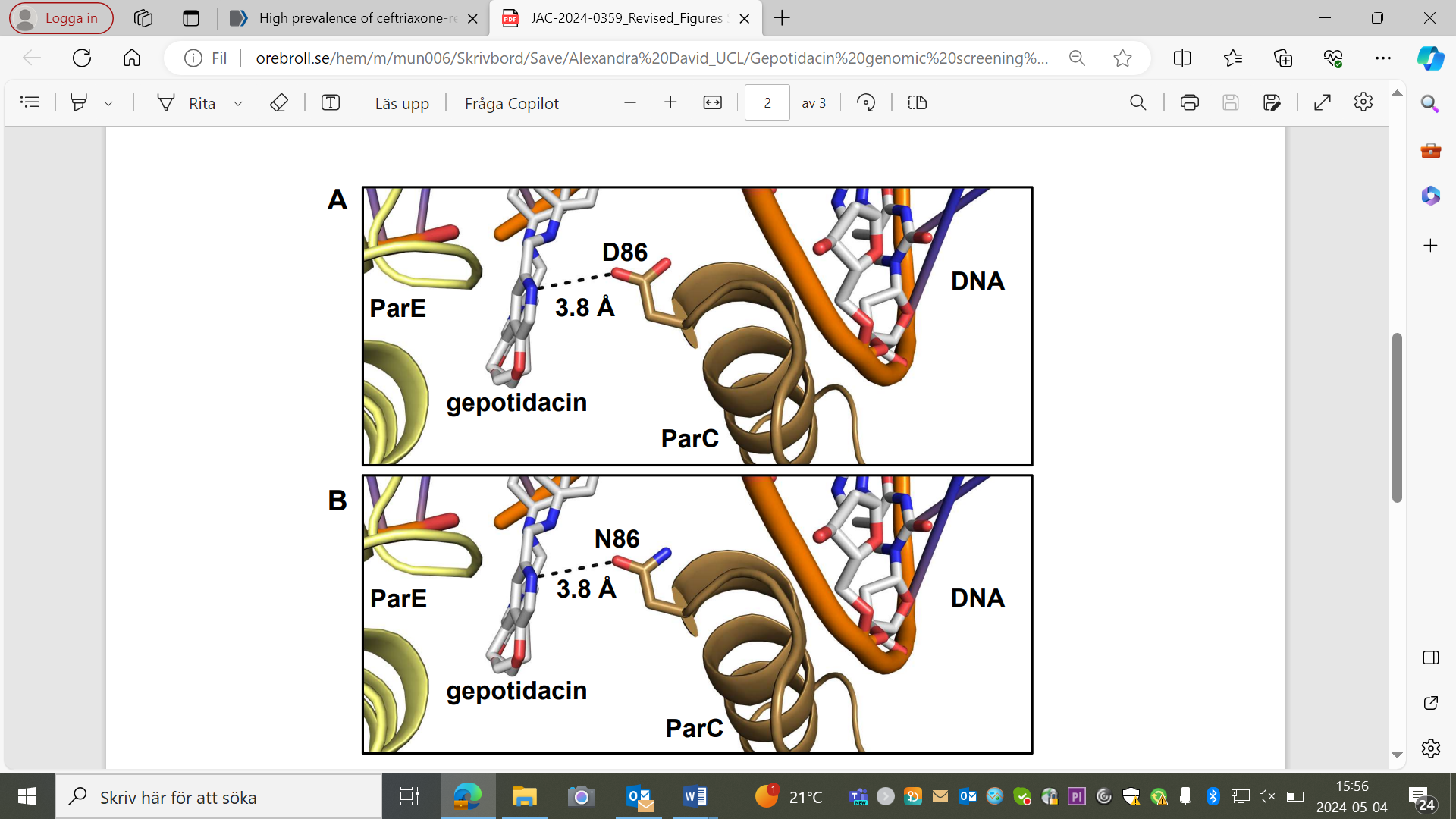


**Figure S2**. The ParC D86N resistance mutation in *Neisseria gonorrhoeae* topoisomerase IV*.* In this model generated by Alphafold, ParE is shown in yellow, ParC in brown and gepotidacin in white. The phosphodiester backbone of DNA is coloured orange and bases are as white bonds. The distance from the Asp or Asn side chains at ParC position 86 to gepotidacin is indicated as a dashed line.


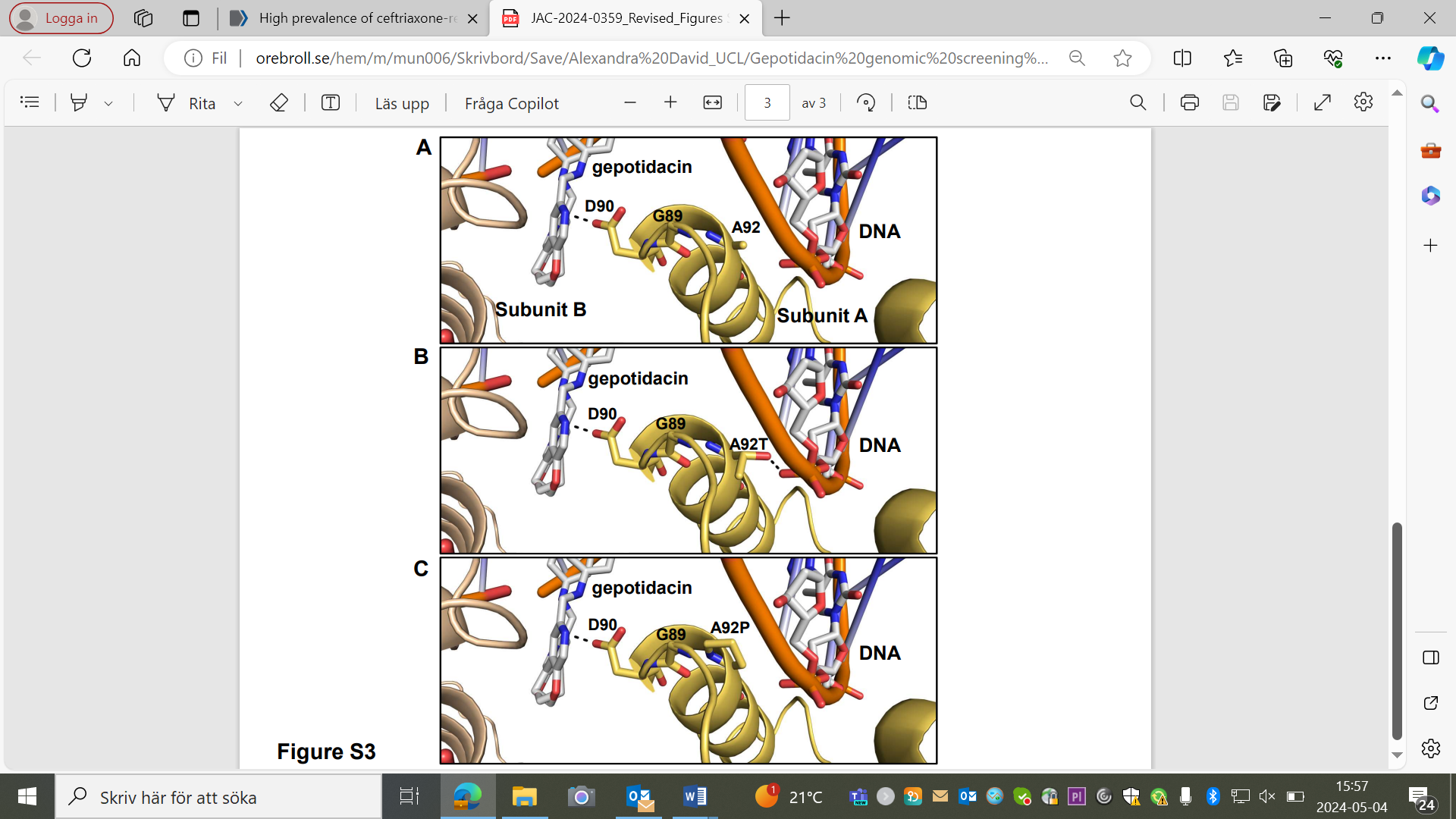


**Figure S3**. *Neisseria gonorrhoeae* GyrA A92 (A), A92T (B) and A92P (C), and their interactions with gepotidacin and DNA. The GyrA subunits A and B are coloured yellow and tan, respectively. DNA is shown with orange backbone and the bases as white bonds. Gepotidacin is shown in white.
